# Recurrent lower respiratory illnesses among young children in rural Kyrgyzstan: overuse of antibiotics and possible under-diagnosis of asthma. A qualitative FRESH AIR study

**DOI:** 10.1038/s41533-018-0081-y

**Published:** 2018-04-10

**Authors:** Marianne Stubbe Østergaard, Jesper Kjærgaard, Mette Marie Kristensen, Susanne Reventlow, Anja Poulsen, Elvira Isaeva, Azamat Akylbekov, Talant Sooronbaev

**Affiliations:** 1 The Research Unit for General Practice and Section of General Practice, Department of Public Health, University of Copenhagen, Copenhagen, Denmark; 2 The Global Health Unit, Department of Paediatrics and Adolescent Medicine, Danish National Hospital “Rigshospitalet”, Copenhagen, Denmark; 3 National Institute of Public Health, University of Southern Denmark, Odense, Denmark; 4 National Center of Maternity and Childhood Care, Bishkek, Kyrgyzstan; 5 National Center of Cardiology and Internal Medicine named after Academician M. Mirrakhimov, Bishkek, Kyrgyzstan

## Abstract

Lower respiratory tract illnesses (LRT-illnesses) in children under 5 years (U5s) are a leading cause of morbidity, hospitalisations and mortality worldwide, particularly in low-income countries. It is pertinent to understand possible inconsistent management. This study explored perceptions and practices among caregivers and health professionals on recurrent LRT-illnesses in U5s. Semi-structured interviews with 13 caregivers to U5s with recurrent LRT-illnesses and with 22 primary care health professional interviews in two rural provinces in Kyrgyzstan were triangulated. Data were thematically analysed. The majority (8/13) of caregivers described their young children as having recurrent coughing, noisy breathing and respiratory distress of whom several had responded positively to acute salbutamol and/or had been repeatedly hospitalised for LRT-illness. Family stress and financial burdens were significant. The health professionals classified young children with recurrent LRT-illnesses primarily with pneumonia and/or a multitude of bronchitis diagnoses. Broad-spectrum antibiotics and supportive medicine were used repeatedly, prescribed by health professionals or purchased un-prescribed by the caregivers at the pharmacy. The health professionals had never applied the asthma diagnosis to U5s nor had they prescribed inhaled steroids, and none of the interviewed caregivers’ U5s were diagnosed with asthma. Health professionals and caregivers shared a common concern for the children’s recurrent respiratory illnesses developing into a severe chronic pulmonary condition, including asthma. In conclusion, the study identified an inconsistent management of LRT-illnesses in U5s, with exorbitant use of antibiotics and an apparently systemic under-diagnosis of asthma/wheeze. When the diagnosis asthma is not used, the illness is not considered as a long-term condition, requiring preventer/controller medication.

## Introduction

Acute respiratory infections are a leading cause of morbidity, hospitalisation and mortality among children under 5 years (U5s) worldwide, particularly in developing countries.^1,2^

Despite the availability of clinical practice guidelines, the management of lower respiratory tract illness (LTR-illness) in young children is often inconsistent^3^ due to challenges in distinguishing between the main diagnostic categories.^4^ Clinically, the diagnostic categories can be divided into: viral infections (e.g., bronchitis, bronchiolitis, viral bronchopneumonia), bacterial infections (e.g., pneumonia, tuberculosis), obstructive diseases (e.g., asthma and viral wheeze) and more uncommon diseases like tracheomalacia and cystic fibrosis. In general, viral infections are self-limiting, while bacterial infections are treatable with antibiotics, and obstructive diseases respond to steroids and bronchodilators.

In young children presenting with LTR-illnesses, former studies have pointed to an over-diagnosis of bacterial pneumonia with overuse of antibiotics and a corresponding under-diagnosis of asthma/viral wheeze.^5–11^ Likewise, U5s with the diagnoses “recurrent pneumonia” or “recurrent bronchitis” often suffered from asthma, indicating that asthma was under-diagnosed and wrongly managed as recurrent pneumonia.^12–15^

The inappropriate use of antibiotics, the growing costs,and an alarming rise in antibiotic microbial resistance pose major health challenges.^16^ World leaders in the G7, G20 and the UN General Assembly have declared antimicrobial resistance to be a global crisis.^17^

Asthma is a high-prevalent heterogeneous disease, likely in young children who have chronic or recurrent long periods of cough, wheeze and/or breathing difficulties, particularly if symptoms are most pronounced at night and in the early morning.^18,19^ However, many young children wheeze with viral infections, and deciding when a child should be given treatment is difficult. The frequency and severity of wheezing episodes and the temporal pattern of symptoms should be taken into account.^19^

Arthur Kleinman emphasised the importance of exploring individual notions of illness in order to understand treatment and health-seeking practices.^20^ Improved LRT-illness care services require a better understanding of caregivers and health professionals' (HPs') perceptions and management of respiratory illnesses. In theoretical support of this, Hanne Mogensen^21^ argued that lay people actively and creatively respond to biomedicine. Farmer^22^ accentuated that the barriers to healthcare seeking are often problems stemming from the weakness of healthcare systems and high costs to the patients in terms of money, time and effort. Farmer assumed that people will use effective and affordable healthcare treatment, if it is available.

This study was conducted as part of the FRESH AIR programme^23^ in two rural provinces in Kyrgyzstan. Kyrgyzstan is a 200,000 square km mountainous country situated in Central Asia. Ethnic Kyrgyz make up the majority of the country’s 6 million inhabitants, of whom 29% are under 15 years of age. Kyrgyzstan attained independence after the breakup of the Soviet Union in 1991. While Kyrgyz is the first language, Russian is retained as a second language, along with strong cultural/scientific relationships. The Kyrgyz healthcare system is predominantly tax financed with universal access. In rural highland districts, primary care operates with HPs in Medical Centres, staffed with family doctors, nurses, paramedics or paediatricians.

The aim of the qualitative study was to explore perceptions and practices among caregivers and HPs on recurrent LRT-illness in U5s and the consequences thereof. Identifying possible inconsistent management is critical to improving healthcare outcomes.

## Results

### Participants

Semi-structured standardised interviews were carried out with 13 caregivers (CGs) (11 mothers, 2 grandmothers) and 22 primary care consulting health professionals (HPs) in public health clinics (7 family physicians, 2 paediatricians, 5 nurses, 8 paramedics). Also, short interviews were carried out with two private pharmacists in Chui. The pilot interviews took place in May 2016 and study interviews were conducted between August and November 2016.

### Perceptions: illness description, terminology and causes

Eight of the 13 interviewed caregivers explained that their child had suffered from recurrent or long-term periods of respiratory illness, with noisy and/or difficult breathing, often peaking at night, lasting weeks or months, which often started in infancy. They used phrases like: constant cough, worsening of cough, wet cough with phlegm, sputum in the bronchi, hurried breathing, noisy or loud breathing, breathing with whistling in the breast, unable to breathe, shortness of breath, and breathing difficulties. Sometimes the illness was accompanied by fever. The respiratory illnesses were frequently in autumn, winter and spring. The expression wheezing was not used by any of the caregivers. The other five children had milder coughs for several periods.

CG1 (Boy 21 months, Naryn): He was already coughing, when I was putting him into cradle. Suddenly I noticed, he was coughing and unable to breathe, turned red… Now the dry cough is constant, shortness of breath occurs seldom… The child doesn’t get well, cough is persistent, no matter what I try.

CG2, (Grandmother, boy 31 months, Naryn): Yes, it happened in February, when for a month or two months there was a shortness of breath and noisy breathing. The child came down with a respiratory illness when he was 1 year old. There were 4-5 episodes of noisy and complicated breathing…. Usually the cough reaches its peak at nights or early in the morning, when the local health posts are closed… Yes, 6 times the child was in the hospital with a cough, sometimes with shortness of breath and increased body temperature.

The HPs responded, when presented with the case history of repeated cough and respiratory distress, that they saw many such cases, several quite severe. A paramedic mentioned around 150 cases a year and a family doctor noted 100 respiratory admissions a year.

HP18 (Paramedic, Chui): Children with chronic bronchitis come, their condition is usually severe: breathing with help of an auxiliary musculature, shortness of breathing, and with normal body temperature.

HP14 (Family doctor, Naryn): There are more admissions with bronchitis and colds. About 100 children during the year and around 3–4 with difficult breathing.

### Fear for prognosis

When asked about the hypothetical prognosis for the children’s LRT-illness, several caregivers and HPs feared a development into disability, respiratory failure, suffocation and death and chronic dependence on medicine. They circled around asthma as a possible prognostic outcome, and some associated asthma with mortality. One mother expressed concern that her child’s condition could turn into tuberculosis.

CG1 (Boy 21 months, Naryn): We are afraid of complications, transition to asthma… Chronic ill, asthma, that child will die.

HP10 (Paramedic, Chui): A child can become disabled… It can pass into a chronic form, or into the bronchial asthma, chronic bronchitis. A little bit cold influence will be enough to manifest these again and again.

### Diagnostic terminology

#### Caregivers’ terms

The caregivers primarily called their child’s recurrent respiratory illness “cough”, “a cold”, “a common cold” or “sore throats”. Several caregivers mentioned different biomedical diagnostic terms used by HPs, like chronic bronchitis and pneumonia. According to the caregivers, the pharmacists, who routinely sold over-the-counter drugs, also called it “a cough”, without using medical diagnostic terms.

CG4 (Boy 49 months, Chui): The obstructive bronchitis was diagnosed twice and the pneumonia was diagnosed twice. He received ceftriaxone and inhaled salbutamol…Pneumonia was established when we were in the hospital…Well, we just call it a cough.

#### Infectious diagnostic terms

When HPs were presented with the case history, alongside their own experiences with U5s with recurrent cough and difficult breathing, the diagnoses “a cold”, “a viral disease”, bronchitis and pneumonia were prevalent. A multitude of *bronchitis* diagnoses with different prefixes were also suggested (Table 1). Some family doctors and paediatricians called it an allergic obstructive bronchitis and related coughing at night to an allergic disease.

**Table 1:** A multitude of bronchitis diagnoses used by health professionals

• Bronchitis
• Simple bronchitis
• Acute bronchitis
• Chronic bronchitis
• Obstructive bronchitis
• Acute obstructive bronchitis
• Bronchitis exacerbations
• Laryngo-tracheo-bronchitis
• Asthmatoid bronchitis
• Allergic bronchitis
• Allergic obstructive bronchitis

However, the diagnosis *pneumonia* prevailed, often unconnected with fever. A family doctor and a paediatrician explained about “protracted courses of pneumonia, bronchitis and viral infections”, another explained that “long-term or recurrent cough can turn into pneumonia”, and one would give a “treatment to prevent pneumonia”.

HP10 (Paramedic, Chui): From the case story: I would call it chronic bronchitis…and prescribe expectorant drug and antibiotics, and Salbutamol if there is a breath difficulty.

HP22 (Family doctor, Chui): Well, if the child is severely coughing at night this is probably bronchitis.

HP1 (Nurse, Naryn): If there is breathing difficulties, chest in drawings of the chest, then that means there is pneumonia; I would prescribe antibiotics.

A few also considered tuberculosis and whooping cough. Some used symptom diagnosis alone or along with diagnostic explanations like “shortness of breath” and “difficult breathing” or “a lot of sputum in the bronchi” or pathophysiological diagnoses like “inflammation of the bronchi, so that they narrow”. One HP referred to the local term “tutok” and explained that it is a respiratory disease caused by exposure to high altitudes.

#### Asthma diagnoses, not used

None of the HPs had diagnosed any U5s with asthma, and some had not even seen any child from 5 to 15 years with asthma. Three doctors and 2 paediatricians said that there may be asthma in young children, but they had not met it. Likewise, none of the interviewed parents had been given the diagnosis asthma to their U5. Paradoxically, most caregivers knew the term asthma. One paediatrician said, “that the parents may think it is asthma and will speculate why the child is not further examined” and another noticed that “some parents complained about wrong diagnoses and insufficient treatment.”

HP3 (Family doctor, Naryn): Asthma in children under 5 years of age may be, but rarely. I did not see it.

HP9 (Paediatrician, Chui): Well, asthma can be in young children. But I have not met.

### Causes and triggers

Among the caregivers, a majority attributed their child´s breathing illnesses to exposure to cold weather and hypothermia.

CG4 (Boy 49 months, Chui): Overcooling, because the children played in cold weather in the irrigation ditch. Moreover, when I am not at home, he walks around the house without socks and slippers.

Other causes mentioned were influenza, not having been breast fed, allergy or heredity. When asked about smoke, caregivers seemed acquainted with the possible relation between smoke and respiratory symptoms, but most did not recall smoke as a triggering factor. None expressed superstitious beliefs, but a few connected the respiratory illness to the child-birth or short duration of breastfeeding.

The HPs emphasised ecological and climatic factors like weather changes and high altitudes, moreover complication of colds due to delayed treatment of cold and flu and viral infections.

HP2 (Nurse, Naryn): There are a lot of admissions from March to April, because in these months the cold and wind begins and in our region all people live in yurts, so it is cold, and the exacerbations are beginning.

A few HPs mentioned smoke from the stove, especially when using dung, but tobacco smoke was not explicitly mentioned as an important trigger. One noticed “allergy to tobacco” as an issue. Others mentioned poor social living conditions or lack of care, malnutrition, lack of vaccinations, allergies or weak immunity.

### Management practices

#### Caregivers’ management and healthcare-seeking practices

The caregivers were determined to act and did not hesitate to use old traditional practices, such as smearing the child’s chest with mutton fat or smearing honey on the child's body or giving the child warm milk with melted butter. The majority mentioned the use of Mukaltin and Herbion syrups to dilute the sputum and Ingalipt spray (sulphonamide) for throat pain and cough. Some caregivers had been on the Internet to get advice such as “legs in hot bath”.

CG3 (Boy 36 months, Chui): Noisy breathing was not there before. And there was a cough, I usually gave Mukaltin and a cough passed. Smeared mutton fat around the neck. … the next day, in the evening, he started a noisy and difficult breathing. I thought that Mukaltin did not help and bought syrup Herbion. On the Internet I looked for what to do in such situations and found that you need to do hot baths on the legs.

Then, often concomitantly or if the condition worsened, the caregivers urgently consulted the local health centre, based on their hitherto confidence in the HPs. Others went directly to the pharmacy. Some went directly to the provincial hospital or even to the national hospital for treatment to shortcut the treatment process, as they expected their HP at the local health centres to refer them to the hospital anyway. Some caregivers complained about wrong diagnoses and ineffective medicine. Alternative practitioners were generally not used.

#### HPs’ management practice

In general, the HPs seemed to be caring and doing their best in the rural health clinics. They provided a wide variety of advice: about keeping warm and warm drinks, about food, like iodised salt, vegetable oil and breastfeeding, and on vitamins to compensate for lack of fresh vegetables. The prescriptions included a multitude of different drugs, often in combinations, including antibiotics, paracetamols, various cough syrups and sometimes antiviral drugs.

Some (a paramedic, a family doctor and the two paediatricians) stated that, if the disease was a viral and self-limiting disease, they would suggest cooling the child, prescribe paracetamol and antiviral drugs, containing interferon alfa (Interferon, Anaferon, Viferon) in the form of suppositories, syrups, tablets or nose drops.

HP9 (Paediatrician, Chui): Now all mums are literate, are engaged in a self-treatment, all time give Amoxicillin. I prescribe Interferon, Anaferon, Viferon and cough syrup, with increasing temperature of paracetamol. I try to treat symptomatically and prescribe antiviral drugs.

#### Antibiotics

Broad-spectrum antibiotics (Amoxicillin, Ampicillin, Streptocid, Amoxiclav and Cephalosporin) were frequently prescribed by a majority of the HPs for U5s presenting with cough and breathing problems. Thus antibiotics were used for the diagnoses bronchitis and pneumonia, with or without stethoscopic rattle, and often regardless of fever, and sometimes preventive, for instance, before referring or before sending the patient for chest X-rays.

Likewise, treatment with un-prescribed antibiotics at the pharmacy was frequently used by caregivers, often copying former prescriptions. Nearly all children had received several therapies with antibiotics, also during hospitalisation. Some caregivers preferred injections of antibiotics, as the antibiotic tablets had not worked.

CG5 (Girl 34 months, Chui): This cough started in August (two months ago), that time the cough was dry and I gave a cough syrup. But there was no effect and we applied to the FOP (health centre), they prescribed amoxicillin… Then a cough appeared again, and a shortness of breath appeared too, there was a noisy breathing, especially at night, and the cough was wet, with the spitting of phlegm.

CG7 (Girl 36 months, Naryn): I usually use Amoxiclav, it helps. When he starts to get sick, I give Amoxiclav.

HP3 (Family doctor, Naryn): If there is a fever, I prescribe antibiotics, Ampicillin, Amoxicillin….

HP13 (Family doctor, Chui): If a child has a cough and shortness of breath, usually depending on the child’s condition, I prescribe antibiotics: Ampicillin intramuscularly… Since children can quickly develop pneumonia, bronchopneumonia. I’m afraid of that.

#### Acute bronchodilators

Inhaled Salbutamol (bronchodilators) were given to children with shortness of breath in the consultation by the family doctors and paediatricians and one paramedic. Caregivers who had experienced their child treated with inhaled salbutamol during hospitalisations noted a high efficacy. None of the caregivers had been prescribed asthma drugs (inhaled bronchodilators nor inhaled corticosteroids) for subsequent use at home.

HP14 (Family doctor, Naryn): I would diagnose the acute obstructive bronchitis…. The first step would be an inhalation of Salbutamol.

CG2 (Grandmother, boy 31 months, Naryn): I know Salbutamol, people used it inhaled through cut bottles and it helped to make his breath free. I am not afraid of this.

#### Referrals and hospitalisations

HPs did not hesitate to refer to a higher level, including hospitals. Most nurses and paramedics in Naryn referred immediately to hospitals, especially regarding children aged <2 years with breathing difficulties. At referral, they often provided antibiotics and painkillers pre-emptively. The paediatricians would refer to an allergist or pulmonologist in case of suspicion of an asthma-like disease. The caregivers, as mentioned, sometimes bypassed the health centre and went directly to a hospital with their children.

Several of the children had been hospitalised >5 times, especially in the cold seasons, and often with “cough”, “shortness of breath” or with the diagnosis of pneumonia or bronchitis. The hospitalisations lasted 5–10 days. According to the caregivers, when admitted to hospitals, the children with respiratory disease were treated routinely with antibiotics, often intramuscularly, apparently often in combination with intramuscular Dexamethasone (steroids) and/or inhaled Salbutamol.

CG11 (Boy 30 months, Chui): But during this year we lay in the hospital with shortness of breath, fever, cough, then we were diagnosed with pneumonia…. we received Ampicillin… Yes, at the hospital he received Salbutamol through the spacer.

### Consequences of long-term illnesses: stress, blame, and systemic barriers

Many parents experienced emotional stress due to uncertainty and confusion about their child’s condition. They were concerned for their child’s present respiratory illness and the future potential disability and even death. Their concerns and questions often went unanswered.

Some parents mentioned guilt, anger and inter-marital problems because of the child’s condition and blamed themselves and each other. Some mothers were blamed for the child’s disease, for not keeping it warm and healthy, or having had problems with the delivery of the baby.

CG5 (Girl 34 months, Chui): My husband and I swear why children get sick so often, maybe we are guilty.

CG4 (Boy 49 months, Chui): Of course, it brings problems. My husband and me, we quarrelled when our child gets sick, and my husband blames me.

Caregivers attributed financial problems to repeated medical expenses as well as frequent health centre visits, referrals and hospitalisations. This led to loss of income due to absence from work. Some identified fruitless visits to local health centres as a reason for going directly to hospitals, despite long distances.

CG2 (Grandmother, boy 31 months, Naryn): In the health center, they recommend some cough syrups, and in case of no effect after a couple of days, I take the child immediately to At-Bashy hospital.

Several HPs mentioned the transport barriers facing caretakers, especially in the mountain region. Additionally, financial difficulties contributed to delayed treatment and complications. Some caretakers even avoided trips to the local health centre for economic reasons. One HP mentioned a 3-year-old child who died because of transportation issues. Other HPs criticised the caregivers for being poorly educated and often postponing the consultations or for not buying the recommended medicine.

HP3 (Family doctor, Naryn): Now, many patients do not go to the doctor, as the financial side is very important…We have no transportation. Weather conditions are not allowing to set out. In the winter, our roads are closed with snow. Snow becomes at the same level with human height. That is why At-Bashy habitants consult doctors late. They die here.

## Discussion

### Main findings

Eight of the 13 interviewed caretakers’ U5s had long-term and/or recurrent coughing, noisy breathing and respiratory distress. Several were hospitalised many times with shortness of breath. The caregivers had adapted a biomedical perception of their child’s illness. They called it mostly “a cold”. The prevailing diagnoses used by the HPs were pneumonia and/or a multitude of bronchitis diagnoses (Table 1). The HPs often managed U5s’ LTR-illnesses with antibiotics, including cough syrups and paracetamols, which also were purchased over-the-counter at the pharmacy by caregivers.

None of the rural HPs had ever diagnosed U5s with asthma nor prescribed inhaled corticosteroids. However, they circled around asthma as a possible prognostic outcome. Many parents experienced emotional stress due to uncertainty and confusion about their child’s present and future condition, the frequent hospitalisations and financial and transport barriers.

### Strengths and limitations

The methodological approach in this study allows for a triangulation of the data collected. Further, the study districts were purposely selected to represent different local conditions. The interviewed family doctors, paediatricians, nurses and paramedics covered a wide spectrum of primary care HPs. This ensured that broad management trends were addressed but did to a lesser degree identify inter-professional nuances and perspectives.

There is a risk of reporting bias, both from the caregivers and HPs, in that they may have provided statements that they think the study staff would like to hear. However, the number of interviews carried out as well as the triangulation of data makes this a lesser risk to the validity of the study. Nuances may have been lost in the translation of data from Kyrgyz to English. However, the data have been translated by Kyrgyz researchers involved in the study.

Recruiting caregivers from health clinics has limited the recruitment base to caregivers who visited the clinic, thus leaving out caregivers who strictly managed their children’s symptoms themselves, or went straight to the hospital.

### Interpretation of findings

The interviews identified several contradictions regarding perceptions, diagnoses and management of children with LRT-illnesses, with numerous deviations from ordinary biomedical practices.

The discussion will focus on apparent overuse of infectious diagnoses, under-diagnosis of asthma/wheeze, severe overuse of antibiotics and not least the consequences.

### Infection diagnoses

The main diagnoses suggested by the HPs, when presented with the case history of a 12-month-old child with recurrent cough and some wheeze, were infectious diagnoses, especially pneumonia and a multitude of bronchitis diagnoses (Table 1).

The prevalent use of the pneumonia diagnosis may have been relevant in some instances. However, the pneumonia diagnosis often disregarded essential criteria, for instance, in children without fever. Furthermore, untraditional labels used such as “protracted courses of pneumonia” are unfounded, as bacterial pneumonia is characterised by acute, high temperature together with cough and respiratory distress and often quickly cured with antibiotics.^24^ In Eigen's study,^12^ 92% of the young children with so-called permanent or recurrent pneumonia had evidence of airway hyper-reactivity, concluding that asthma was a common cause, even in the absence of wheezing.

The variety of bronchitis diagnoses applied (Table 1) were often managed with antibiotics. However, bronchitis and “acute bronchitis” are defined as virally induced and self-limiting infections with cough,^25^ without the need for antibiotics. Likewise, the diagnosis “chronic bronchitis” signals a long-term infection, which in other settings is associated with asthma.^26^ Other bronchitis prefixes used, such as: “obstructive”, “asthmatic”, and “allergic” or “allergic obstructive bronchitis” connote to viral asthma/wheeze. However, the treatment provided was mostly antibiotics, which is not aligned with the management of recurrent viral wheeze/asthma in U5s.^27,28^

### Under-diagnosis of asthma/viral wheeze

None of the interviewed HPs had ever applied the diagnosis asthma to young children. This is despite of the ISAAC study findings of an asthma prevalence of 12.2% in Kyrgyzstan among 6–7-year-old school-children^29^ and despite the onset of asthma often emerging in infancy.^18^

Likewise, none of the caregivers’ U5s were diagnosed with asthma. The characteristic of the U5s LTR-illnesses, described by more than half of the caregivers, were recurrent and/or long-term periods of cough, noisy breathing (wheezing) and shortness of breath, often peaking at night. This symptom pattern and severity is compatible with asthma criteria in U5s.^18,19^ Several of the U5s had received inhaled bronchodilators acutely in the health centres or in hospitals for shortness of breath, with high efficacy, which supports the asthma diagnosis.^18,19^ Also, the symptoms often followed colds in the autumn and winter, which fits with viral respiratory tract infections being a predominant trigger of asthma exacerbations.^30,31^

Paradoxically, most caregivers knew about the diagnostic term asthma, and HPs and caregivers hovered around asthma as a possible outcome following the U5s' recurrent LTR-illness, possibly due to the HPs' tacit acknowledgement of recurrent respiratory illnesses possibly developing into asthma.

### Explanatory models of illness

Kleinman’s theory of explanatory models of illness argued that practitioners organise knowledge in mental categories of prototypical events or cognitive/cultural models, influenced by former knowledge and the present situation.^20^ In this study, the HPs' internalised patterns of cultural tradition in rural Kyrgyz did not include the asthma diagnosis in young children, and the HPs seemed entrenched in their assumptions that “no young children in the village had asthma”.

So, unappreciated asthma may have been the case in this Kyrgyz setting, in accordance with former findings.^12–15^ The design of this study did not allow us to determine how frequently asthma was under-diagnosed.

### Over-prescription of antibiotics

Broad-spectrum antibiotics seemed widely prescribed by the local HPs, except for the two interviewed paediatricians. Moreover, antibiotics were repeatedly purchased over-the-counter at the pharmacy by the caregivers. However, the effect of antibiotics for LTR-illnesses, when bacterial pneumonia is not suspected, is at best moderate,^32,33^ indicating that a large amount of the antibiotic prescriptions seemed inappropriate.

Antibiotic prescribing is directly linked to the development of antibiotic resistance, emphasising the need to minimise use and avoid misuse of antibiotics.^16^ The over-prescribing of antibiotics depends on factors such as physicians’ diagnostic uncertainty, caregivers’ expectations of receiving antibiotics and physicians’ perception of what caregivers want.^34,35^

The apparent “missing diagnosis” of asthma for young children in the two rural provinces in Kyrgyzstan probably contributed to antibiotic over-prescribing, due to diagnostic inconsistencies and incorrect associations between substitute diagnoses and antibiotics. The problem of over-use of antibiotics is likely to be even more pronounced in areas of the world with unregulated, over-the-counter sale of antibiotics.^36^

### Systemic barriers and consequences

The diagnostic inconsistencies and substitute diagnoses seemed to result in a situation of a collective heavy use of health resources, including much medicine and self-medication via the pharmacy, referrals, emergency visits and long-lasting hospitalisation at provincial or national paediatric hospitals. As their child’s illness kept recurring, caregivers experienced fear, uncertainty and doubt. Despite substantial financial and transport challenges, especially in isolated highland locations, some bypassed the health centres and went directly to hospitals, because of prior, fruitless visits to local health centres. This is in line with Lynch’s study, showing that children with a delay in asthma diagnosis were more likely to visit emergency care centres at least once.^37^

In general, the caregivers were alert, active and pragmatic in handling their children’s disease and were in general satisfied with the help from the local health centre. They combined self-treatment with a help-seeking practice at the local health centre or directly to a hospital. This is in line with Mogensen’s study^21^ arguing that caregivers felt responsible and open to biomedicine.

This pragmatic approach is supported by Farmer´s claim that people will seek help if and where help is available.^38,39^ Farmer^22^ related healthcare seeking, availability, affordability and quality of healthcare to successful healthcare outcomes and demonstrated that active identification of the systemic, structural obstacles is vital to getting necessary help to those who are ill and socio-economically disadvantaged.

### Conclusions and implications

The study identified challenges and paradoxes in handling U5s with recurrent LRT-illnesses in two rural provinces in Kyrgyzstan, indicating that the prevailing diagnoses, pneumonia and a multitude of bronchitis diagnoses, have at times been under-appreciated asthma/wheeze.

When the diagnosis asthma/wheeze is not used, the respiratory illness is not considered as a long-term condition, requiring preventer/controller medication. The diagnostic inconsistencies apparently created a situation of collective overuse of antibiotics, repeated consultations, referrals and hospitalisations. This amplified family financial and transport burdens, especially in the rural highland districts.

To reduce the apparent systemic under-diagnosis of asthma and the emerging antibiotic resistance, adequate training in LTR-illnesses and management for asthma/wheeze can be an essential contribution. Moreover, a stricter supervision of the sale of antibiotics in retail pharmacies probably reduces over-use of antibiotics.

## Methods

This study has been conducted as a qualitative study, relying on the principles of qualitative data-collection and analysis under the COREQ consensus statement.^40^

### Settings

Data collection for the study was conducted in health clinics in two different provinces in Kyrgyzstan, one in the lowlands (Chui province) close to the capital Bishkek and one in the highlands (Naryn province), far from the capital. The administrative leader and the healthcare professionals were contacted beforehand and asked for permission and participation.

### Interviews and topic guide

Semi-structured interviews were carried out with caregivers and primary care consulting HPs. The topic guide (Table 2) and the standardised interview guide were inspired by a set of questions formulated by Arthur Kleinman (Table 3) to elicit individual explanatory models of illness^20^ and by theoretical perspectives on healthcare availability by Paul Farmer.^22^ At the start of the interview, caregivers were asked to describe their child’s illness and the management from the beginning. Likewise, HPs were presented with a case history concerning a 12-month-old child with recurrent episodes of cough and noisy breathing lasting >2 weeks and were asked about their experience managing U5s with recurrent respiratory illness. The interview guides were translated into Russian by local researchers and pilot tested by cooperation between the Kyrgyz and Danish research team.

**Table 2:** Topic guide and coding framework for recurrent LTR-illness in U5s

Topic guide	Coding framework
Background information	HPs’ background
	Caregivers’ background
Following the case history: HPs’ experiences with recurrent LTR-illnesses	HPs’ experiences with similar respiratory illnesses in U5s and their diagnostic practices
The child’s respiratory disease history	The child’s respiratory disease history (debut, symptoms, duration)
The terms, common words and concepts for cough and resp. distress	Caregivers’ name for the child’s disease
	HPs’ diagnostic terminology
	HPs’ names/diagnoses given to caregivers for the child's repeated cough and respiratory distress
Explanations (causes) and severity of respiratory symptoms	Causes
	Triggers
	Severity
Prognostic thoughts	HPs’ prognostic thoughts
	Caregivers’ prognostic thoughts
Management practice	HPs’ advice and information
	HPs’ treatment
	HPs’ problems experienced in treating children
	HPs’ referrals
	Caregivers’ self-management
	Caregivers’ help-seeking—when?
	Caregivers’ help-seeking—where?
	Treatment at the local health centre
	Treatment at hospitals
Caregivers’ problems, meaning, beliefs, fears, stigma	Problems the coughing sickness has caused in the family
	Meaning, belief and fear in the family
	Community belief/stigmas/ humiliating stereotype
Satisfaction and Barriers	Satisfaction with help-seeking
	Barriers to healthcare seeking (financial, accessibility, affordability)

**Table 3:** Arthur Kleinman’s 8 questions

1. What do you call the problem?
2. What do you think has caused the problem?
3. Why do you think it started when it did?
4. What do you think the sickness does? How does it work?
5. How severe is the sickness? Will it have a long or a short duration?
6. What kind of treatment do you think the patient should receive?
7. What are the chief problems the sickness has caused?
8. What do you fear most about the sickness?

### Inclusion and recruitment criterion

The inclusion criterion for the participant caregivers was having a child aged between 12 and 59 months with long-term and/or recurrent cough visiting a health clinic for LTR-illness, without a diagnosis of tuberculosis. Caregivers were selected by a purposive sampling, aiming for variations in health clinic locations (by recruiting in seven different clinics in Naryn and four clinics in Chui), age and numbers of children. Likewise, the HPs were purposely sampled with regard to different local health centres, all recruited in different villages in the two provinces, and different educational levels (family doctors, paediatricians, paramedics, nurses).

### Data collection

The pilot interviews took place in May 2016 in cooperation between the Kyrgyz and Danish research team. The rest of the interviews were carried out between August and November 2016 by the Kyrgyz team, bilingual in Kyrgyz and Russian, in the respondent’s native language and lasted approximately 1 h. Debriefing matrices for each interview were made shortly after the interviews. Field notes were taken during and after observations and interviews. The interviews were audiotaped, transcribed and translated into English.

### Data analysis and interpretation

The data were analysed for concepts following a coding strategy described by Corbyn and Strauss.^41^ A thematic coding framework,^42^ deductively based on the topic guide (Table 2), was generated for the key themes and subthemes. First, an open reading of the raw data was conducted by several members of the study team. Manual coding of the data was conducted using a coding tree. For coding, each transcript was read and re-read by two researchers several times to understand and decide on allocation of codes.

### Ethical consideration

The study was approved by the Ethical and Research Committee in Kyrgyzstan and was conducted according to the ethical guidelines of the Helsinki Declaration. The research complied with local ethical regulations. Written consent was obtained from all patients.

### Data availability

Access to the data sets supporting the conclusions of this manuscript may be obtained upon reasonable request from the author.
