# Author Correction: Recurrent lower respiratory illnesses among young children in rural Kyrgyzstan: overuse of antibiotics and possible under-diagnosis of asthma. A qualitative FRESH AIR study

**DOI:** 10.1038/s41533-018-0082-x

**Published:** 2018-06-28

**Authors:** Marianne Stubbe Østergaard, Jesper Kjærgaard, Mette Marie Kristensen, Susanne Reventlow, Anja Poulsen, Elvira Isaeva, Azamat Akylbekov, Talant Sooronbaev

**Affiliations:** 1 The Research Unit for General Practice and Section of General Practice, Department of Public Health, University of Copenhagen, Copenhagen, Denmark; 2 The Global Health Unit, Department of Paediatrics and Adolescent Medicine, Danish National Hospital “Rigshospitalet”, Copenhagen, Denmark; 3 National Institute of Public Health, University of Southern Denmark, Odense, Denmark; 4 National Center of Maternity and Childhood Care, Bishkek, Kyrgyzstan; 5 National Center of Cardiology and Internal Medicine named after Academician M. Mirrakhimov, Bishkek, Kyrgyzstan

**Correction to:** *npj Primary Care Respiratory Medicine* (2018) 10.1038/s41533-018-0081-y; published online 10 April 2018

The original version of this article contained an error in the spelling of the author Mette Marie Kristensen, which was incorrectly given as Mette-Marie Kristensen. The affiliation details for Mette Marie Kristensen were also incorrect in this Article. The correct affiliation details for this author are given below:

National Institute of Public Health, University of Southern Denmark, Odense, Denmark

This has now been corrected in both the PDF and HTML versions of the Article.

